# Ward-based clinical pharmacists and hospital readmission: a non-randomized controlled trial in Sri Lanka

**DOI:** 10.2471/BLT.17.198366

**Published:** 2017-11-30

**Authors:** Lelwala Guruge Thushani Shanika, Shaluka Jayamanne, Chandrani Nirmala Wijekoon, Judith Coombes, Dhineli Perera, Fahim Mohamed, Ian Coombes, Hithanadura Asita De Silva, Andrew Hamilton Dawson

**Affiliations:** a Department of Allied Health Science, Faculty of Medical Sciences, University of Sri Jayewardenapura, Nugegoda, Sri Lanka.; b Faculty of Medicine, University of Kelaniya, Ragama, Sri Lanka.; c Faculty of Medical Sciences, University of Sri Jayewardenapura, Nugegoda, Sri Lanka.; d School of Pharmacy, University of Queensland, Brisbane, Australia.; e Department of Pharmacy, Austin Health, Melbourne, Australia.; f Faculty of Medicine, University of Peradeniya, Peradeniya, Sri Lanka.; g Faculty of Medicine, University of Kelaniya, Ragama, Sri Lanka.; h Faculty of Medicine, University of Sydney, Sydney, Australia.

## Abstract

**Objective:**

To assess if a ward-based clinical pharmacy service resolving drug-related problems improved medication appropriateness at discharge and prevented drug-related hospital readmissions.

**Method:**

Between March and September 2013, we recruited patients with noncommunicable diseases in a Sri Lankan tertiary-care hospital, for a non-randomized controlled clinical trial. The intervention group received usual care and clinical pharmacy service. The intervention pharmacist made prospective medication reviews, identified drug-related problems and discussed recommendations with the health-care team and patients. At discharge, the patients received oral and written medication information. The control group received usual care. We used the medication appropriateness index to assess appropriateness of prescribing at discharge. During a six-month follow-up period, a pharmacist interviewed patients to identify drug-related hospital readmissions.

**Results:**

Data from 361 patients in the intervention group and 354 patients in the control group were available for analysis. Resolutions of drug-related problems were higher in the intervention group than in the control group (57.6%; 592/1027, versus 13.2%; 161/1217; *P* < 0.001) and the medication was more appropriate in the intervention group. Mean score of medication appropriateness index per patient was 1.25 versus 4.3 in the control group (*P* < 0.001). Patients in the intervention group were less likely to be readmitted due to drug-related problems (44 patients of 311 versus 93 of 311 in the control group; *P* < 0.001).

**Conclusion:**

A ward-based clinical pharmacy service improved appropriate prescribing, reduced drug-related problems and readmissions for patients with noncommunicable diseases. Implementation of such a service could improve health care in Sri Lanka and similar settings.

## Résumé

**Objectif:**

***Évaluer si la présence dans les étages d'un service de pharmacie clinique résolvant les problèmes liés aux médicaments a permis de mieux adapter les prescriptions médicales à la sortie de l'hôpital et d'empêcher les réadmissions dues aux médicaments.***

**Méthodes:**

***Entre mars et septembre 2013, nous avons recruté des patients atteints de maladies non transmissibles dans un hôpital de soins tertiaires du Sri Lanka afin de participer à un essai clinique contrôlé non randomisé. Le groupe expérimental a bénéficié de la prise en charge habituelle et d'un service de pharmacie clinique. Le pharmacien de l'essai a fait un examen prospectif des traitements, a repéré les problèmes liés aux médicaments et a discuté des recommandations avec l'équipe de soins et les patients. À leur sortie de l'hôpital, les patients ont été informés oralement et par écrit au sujet du traitement prescrit. Le groupe témoin a bénéficié de la prise en charge habituelle. Nous avons utilisé le Medication Appropriateness Index (indice de pertinence des traitements) pour évaluer la pertinence des prescriptions à la sortie de l'hôpital. Pendant une période de suivi de six mois, un pharmacien a interrogé les patients afin de déterminer si les réadmissions à l'hôpital étaient dues à des médicaments.***

**Résultats:**

***Nous avons pu analyser les données de 361 patients du groupe expérimental et de 354 patients du groupe témoin. Les problèmes liés aux médicaments ont été davantage résolus dans le groupe expérimental que dans le groupe témoin (57,6%; 592/1027, contre 13,2%; 161/1217; P < 0,001) et les traitements étaient plus adaptés dans le groupe expérimental. Le score moyen par patient du Medication Appropriateness Index était de 1,25 contre 4,3 dans le groupe témoin (P < 0,001). Les patients étaient moins nombreux à être réadmis pour des problèmes liés à des médicaments dans le groupe expérimental (44 patients sur 311 contre 93 sur 311 dans le groupe témoin; P < 0,001).***

**Conclusion:**

***La présence d'un service de pharmacie clinique dans les étages a permis d'améliorer la pertinence des prescriptions et de réduire les problèmes liés aux médicaments ainsi que les réadmissions pour les patients atteints de maladies non transmissibles. La mise en place d'un service de ce type pourrait améliorer la prise en charge médicale au Sri Lanka et dans des régions similaires.***

## Resumen

**Objetivo:**

Evaluar si un servicio de farmacia clínica en planta para la resolución de los problemas relacionados con los medicamentos, mejoró la adecuación de la medicación en el momento del alta y evitó las readmisiones hospitalarias relacionadas con los medicamentos.

**Métodos:**

Entre marzo y septiembre de 2013, captamos pacientes con enfermedades no contagiosas en un hospital de atención terciaria de Sri Lanka, para un ensayo clínico no aleatorizado controlado. El grupo de intervención recibió la atención habitual y servicios de farmacia clínica. El farmacéutico de intervención realizó las revisiones prospectivas de la medicación, identificó los problemas relacionados con los medicamentos y discutió las recomendaciones con el equipo de atención médica y los pacientes. En el momento del alta, los pacientes recibieron información oral y escrita sobre los medicamentos. El grupo de control recibió la atención habitual. Usamos el índice de adecuación de la medicación para evaluar la adecuación de la prescripción en el momento del alta. Durante un periodo de seguimiento de seis meses, un farmacéutico entrevistó a los pacientes para identificar las readmisiones hospitalarias relacionadas con los medicamentos.

**Resultados:**

Los datos de 361 pacientes en el grupo de intervención y de 354 pacientes en el grupo de control estuvieron disponibles para el análisis. Las resoluciones de los problemas relacionados con los medicamentos fueron más altas en el grupo de intervención que en el grupo de control (57,6%; 592/1027, frente a 13,2%; 161/1217; *P* < 0,001) y la medicación fue más adecuada en el grupo de intervención. La puntuación media del índice de adecuación de la medicación por paciente fue de 1,25 frente a 4,3 en el grupo de control (*P* < 0,001). Los pacientes en el grupo de intervención tenían menos probabilidades de ser readmitidos a causa de problemas relacionados con la medicación (44 pacientes de 311 frente a 93 de 311 en el grupo de control, *P* < 0,001).

**Conclusión:**

Un servicio de farmacia clínica en planta mejoró la prescripción adecuada de medicamentos, redujo los problemas relacionados con los medicamentos y las readmisiones de pacientes con enfermedades no contagiosas. La implementación de dicho servicio podría mejorar la atención médica en Sri Lanka y en entornos similares.

## ملخص

**الغرض:**

تقييم دور خدمات الصيدلة العلاجية المقدمة للمرضى النزلاء بالمستشفيات بما تهدف إليه من حل المشكلات المتعلقة بالدواء وحقيقة إسهام تلك الخدمات في رفع مستوى ملاءمة الأدوية الموصوفة للمرضى عند خروجهم من المستشفى وتفادي عودتهم من جديد للاستشفاء داخل المستشفى.

**الطريقة:**

استعنا بمرضى مصابين بأمراض غير معدية داخل مستشفى للعلاج التخصصي في سري لانكا، في الفترة بين مارس/آذار وسبتمبر/أيلول من عام 2013، وذلك لإجراء تجربة سريرية غير معشاة مضبطة بالشواهد. وتلقت المجموعة التجريبية الرعاية الطبية والخدمة الصيدلانية العلاجية بالطريقة المعتادة. وأجرى الصيدلي المختص بالتدخل العلاجي مراجعات استباقية للأدوية الموصوفة، وحدد المشكلات المتعلقة بالدواء، وناقش التوصيات مع كل من فريق الرعاية الصحية والمرضى. وعند خروج المرضى من المستشفى، تلقوا معلومات شفهية ومكتوبة بشأن الأدوية. وحصلت مجموعة المقارنة على الرعاية الصحية المعتادة. واستعنا بمؤشر ملاءمة الدواء لتقييم مدى كفاءة وصف الأدوية للمرضى عند خروجهم من المستشفى. والتقى أحد الصيادلة مع المرضى خلال فترة متابعة استمرت لستة أشهر، للوقوف على حالات عودة المرضى للاستشفاء من جديد داخل المستشفيات لأسباب تتعلق بالأدوية.

**النتائج:**

توفرت بيانات مستمدة من 361 مريضًا يندرجون ضمن المجموعة التجريبية و354 مريضًا يندرجون ضمن مجموعة المقارنة، وخضعت تلك البيانات للتحليل. وارتفعت نسبة التوصل لحلول للمشكلات المتعلقة بالدواء في المجموعة التجريبية مقارنةً بمجموعة المقارنة (57.6‏%؛ 592‏/1027، في مقابل 13.2‏%؛ 161/1217؛ *بنسبة احتمال * < 0.001) وكان الدواء أكثر ملاءمة للمرضى في المجموعة التجريبية، إذ بلغ متوسط نتائج مؤشر ملاءمة الدواء للمريض الواحد 1.25 في مقابل 4.3 لدى المرضى في مجموعة المقارنة (بنسبة احتمال < 0.001). وانخفضت في المجموعة التجريبية احتمالية عودة المرضى للاستشفاء من جديد داخل المستشفى بسبب مشكلات تتعلق بالأدوية (فكانت النسبة 44 من بين 311 في مقابل 93 من بين 311 في مجموعة المقارنة؛ *بنسبة احتمال * < 0.001).

**الاستنتاج:**

أدت خدمات الصيدلة العلاجية المقدمة للمرضى النزلاء بالمستشفيات إلى ارتفاع كفاءة وصف الدواء الملائم للمرضى، مما أدى إلى تقليل المشكلات المتعلقة بالأدوية وحالات عودة المرضى المصابين بأمراض غير معدية للاستشفاء من جديد داخل المستشفيات. إن تقديم هذا النوع من الخدمات قد يؤدي إلى تحسين الرعاية الصحية في سري لانكا والأوساط المشابهة لها. ****

## 摘要

**目的:**

旨在评估基于病房的临床配药服务解决与药物相关的问题后是否能够在出院时改善药物治疗的适宜性并预防与药物相关的返院治疗。

**方法:**

我们于 2013 年 3 月到 9 月期间在斯里兰卡三级护理医院招募了非传染性疾病患者，进行了非随机对照临床试验。干预组接受了常规护理和临床配药服务。干预药剂师开展了具有前瞻性的药物评审，确定了与药物有关的问题，并与医疗护理团队和患者讨论了建议。出院时，患者收到了口头和书面的医疗信息。对照组接受了常规护理。我们采用了药物适宜性指标来评估出院时开药的适宜性。在六个月的随访期内，药剂师对患者进行访问以确定与药物相关的返院治疗问题。

**结果:**

来自干预组 361 位患者和对照组 354 位患者的数据可供分析。与对照组相比，干预组解决与药物有关问题的概率更高（57.6%；592/1027，对比 13.2%；161/1217；P < 0.001），干预组的药物更适宜。患者药物适宜性指标的平均分数分别为 1.25 和 4.3（对照组）(P < 0.001)。干预组患者由于与药物相关的问题而返院治疗的可能性更低（311 位患者中有 44 位，对照组 311 位患者中有 93 位；P < 0.001）。

**结论:**

基于病房的临床配药服务提升了处方适宜性，减少了非传染性疾病患者与药物相关的问题和返院治疗率。此类服务的实施能够提升斯里兰卡及类似地区的医疗护理水平。

## Резюме

**Цель:**

Оценить, способствовала ли деятельность больничной клинической аптечной службы по разрешению проблем, связанных с лекарственными препаратами, повышению частоты рационального назначения лекарственных препаратов при выписке и предотвращению повторной госпитализации в связи с приемом назначенных лекарственных препаратов.

**Методы:**

В период с марта по сентябрь 2013 года в высокоспециализированной больнице в Шри-Ланке мы приглашали пациентов с неинфекционными заболеваниями для участия в нерандомизированном контролируемом клиническом исследовании. Экспериментальная группа получила стандартное медицинское и аптечное обслуживание. Фармацевт провел проспективный обзор назначенных лекарственных препаратов, выявил связанные с ними проблемы и обсудил рекомендации со службой здравоохранения и пациентами. При выписке пациенты получили информацию о лечении в устном и письменном виде. Контрольная группа получила только стандартное медицинское обслуживание. Мы использовали индекс приемлемости лекарственных препаратов для оценки рациональности их назначения при выписке. В течение шестимесячного периода последующего наблюдения фармацевт проводил опросы пациентов для выявления случаев повторной госпитализации, связанной с приемом назначенных лекарственных препаратов.

**Результаты:**

Для анализа были доступны данные 361 пациента в экспериментальной группе и 354 пациентов в контрольной группе. Разрешение проблем, связанных с приемом назначенных лекарственных препаратов, было выше в экспериментальной группе по сравнению с контрольной группой (57,6%, 592/1027, против 13,2%, 161/1217, *Р* < 0,001), и назначение лекарственных препаратов было более рациональным в экспериментальной группе. Среднее значение показателя приемлемости лекарственного средства в расчете на одного пациента составило 1,25 против 4,3 в контрольной группе (*P* < 0,001). Пациенты в экспериментальной группе имели меньшую вероятность повторной госпитализации из-за проблем, связанных с приемом назначенных лекарственных препаратов (44 пациента из 311 против 93 из 311 в контрольной группе, *P* < 0,001).

**Вывод:**

Деятельность больничной клинической аптечной службы повысила частоту рационального назначения лекарственных препаратов, уменьшила возникновение проблем, связанных с приемом назначенных лекарственных препаратов, и частоту повторной госпитализации пациентов с неинфекционными заболеваниями. Внедрение такой службы может улучшить работу системы здравоохранения в Шри-Ланке и в аналогичных условиях.

## Introduction

Noncommunicable diseases, such as cardiovascular diseases, cancer, chronic respiratory diseases and diabetes, are a major global health burden and are an increasing problem in low- and middle income countries.^1^^,^^2^ An important component of care for noncommunicable diseases is the quality use of medicines – that is, selecting management options wisely, choosing suitable medicines if a medicine is considered necessary and using medicines safely and effectively. Failure of following this component may lead to drug-related problems, which are defined as “an event or circumstance involving drug therapy that actually or potentially interferes with desired health outcomes.”^3^ Such problems cause a large number of hospital admissions, many of which are avoidable.^4^

In high-income countries, clinical pharmacy services have been shown to improve quality use of medicines and reduce drug-related problems, hospital readmissions and health-care expenditures.^5^^–^^8^ In low- and middle-income countries clinical pharmacy services are limited^9^^,^^10^ and implementation of such services could faces various challenges, such as: (i) lack of clinically qualified pharmacists;^11^ (ii) poor pharmaceutical literacy among patients;^12^ (iii) under-utilization of research evidence due to underdeveloped health-care systems;^13^ (iv) restrictions in medicines regulatory capacity;^13^ (v) poor availability of essential medicines; and (vi) limitations in accessing high quality medicines.^11^ Hence, findings from implementation of clinical pharmacy services in high-income countries cannot be generalizable to low- and middle-income countries.

In Sri Lankan public hospitals, there are no established ward-based clinical pharmacy services. Hospital pharmacists are engaged in procurement, storage and dispensing of medicine to patients. However, we have previously identified opportunities for clinical pharmacists to contribute to the quality use of medicines.^14^

This study evaluates the impact of a clinical pharmacy service in a Sri Lankan hospital. We assessed the resolution of inpatient drug-related problems, appropriateness of medications at discharge from hospital and drug-related hospital readmissions.

## Method

### Study setting and design

We recruited patients from March to September 2013 for this non-randomized controlled clinical trial and made a follow-up from October 2013 to March 2014. The study took place in the university medical unit of the Colombo North Teaching Hospital, Sri Lanka, a 1421-bed tertiary-care hospital. This unit has a female and a male ward of 55 and 65 beds, respectively.

We recruited patients from the two medical wards for the intervention and control groups and we allocated wards instead of patients within wards to reduce the risk of group contamination. Between March and May 2013, the patients in the male ward received the intervention and the female patients were allocated to the control group. In June, none of the wards received the intervention. Between July and September 2013, the female patients received the intervention and male patients were allocated to the control group.

### Study population

Eligible patients were those with chronic noncommunicable diseases who needed long-term follow-up at the medical clinic.^1^ Each day of the recruitment period, an independent medical officer approached eligible patients in each ward, as recorded chronologically in the admission register, informed them about the study and the voluntarily enrolment, and continued until five patients were recruited.

The patients also received an information leaflet in their native language. For patients agreeing to participate, they gave both a written and a verbal consent to be seen and followed-up by a pharmacist. We excluded patients if they had impaired conscious level with no carer to manage medicines, receiving long-term follow-up from another unit or had communication difficulties.

Using a 50% increase in patient knowledge after discharge as a surrogate for improved drug use, we calculated a sample size was 400 patients for each arm with the level of significance of 0.05 and statistical power of 90%.

### Intervention

In addition to the standard care provided by physicians and nurses, the intervention group received clinical pharmacy services from a clinical intervention pharmacist, who recorded the current medication history of the patient on admission and reviewed the patients’ medication charts daily until discharge. To optimize the patient’s medications, the intervention pharmacist reconciled the medication history taken at admission with the physician’s admission medication history. Then at the time of transfer or discharge the prescribed medications were again reviewed. Discrepancies including deletions, additions or changes to the medication lists were noted. The pharmacist identified potential drug-related problems, recorded them on a data collection form and discussed the findings and possible resolutions with the health-care team at any time during the admission, hospital stay and at the time of discharge. For problems related to patient’s knowledge about the medication, the pharmacist discussed the problem with the patient and gave recommendations. At discharge, the pharmacist provided the patients with verbal and written instructions, given in the local language, on the safe administration of their medicines and a medicine list. Providing this type of education at ward level was not current practice in the Sri Lankan hospital system.

Patients in the control group received only the standard care. An assessment pharmacist interviewed the control group patients to obtain their medication history at discharge and reviewed their pharmacotherapy retrospectively to identify drug-related problems. This pharmacist did not provide any feedback to attending medical teams and did not provide any education during hospital stay or at discharge.

The two pharmacists were not involved in recruitment of participants, but they were aware of the group allocation. Both pharmacists were local graduates with a Sri Lankan Bachelor of Pharmacy degree, who received training in clinical pharmacy as part of their undergraduate curriculum and were employed specifically for the study. At one occasion before the study and two occasions during the study, the pharmacists received local ward-based teaching from external senior Australian clinical pharmacists. This training included reviewing of medications and refining data collection and patient interview techniques. Once recruitment began, the senior clinical pharmacists located in Australia held weekly case-based tutorials and quality checked the medication reviews, via the telecommunication software Skype (Microsoft, Redmond, United States of America).^14^

### Outcomes

Daily, the assessment pharmacist independently categorized the identified drug-related problems into one of six main classes – adverse drug reaction, dosing error, wrong medicine or no medicine, drug use problem, interaction and other – according to the Pharmaceutical Care Network Europe classification scheme version 5.01.^3^ Every fortnight, in a Skype meeting, the two pharmacist, the two senior pharmacists and two local consultant physicians validated the categorization of selected drug-related problems (1167/2244). They reached consensus for any discrepancy by discussion and they recategorized drug-related problems when necessary.

The intervention pharmacist recorded the outcomes of the discussions with either health-care team or patients on a data collection form as either accepted – that is, when the health-care team and patients accepted the recommendations – or not accepted, that is, when the health-care team and patients did not agree with the recommendations. The assessment pharmacist received the data collection form after the patient was discharged and further classified accepted recommendations into either implemented or not implemented. Problems deemed not to be clinically important by the intervention pharmacist were not brought to the attention of the health-care team and were recorded as not discussed. Patients with resolved drug-related problems, either in the intervention group or control group, without the pharmacist’s intervention were recorded as self-resolved. A more detailed description of the acceptance and attitudes of health-care staff members towards the introduction of clinical pharmacy service is reported elsewhere.^15^

To evaluate the appropriateness of the prescribed medications at discharge, the assessment pharmacist used the validated tool: medication appropriateness index.^16^ The index has judgment-based and criterion-based measures.^16^ Summated index scores for a medication range from 0 to 18 with lower scores reflecting a more appropriate medication.^17^ A patient score is the mean score of all the patients’ medications. We categorized those patients with a score of zero for all their medications as receiving appropriate medications.

After discharge, the assessment pharmacist interviewed the participants monthly, for a period of six months, to identify any drug-related hospital readmissions. The pharmacist used a pre-tested questionnaire to document readmission information. The pharmacist identified possible causes for drug-related readmissions as either adverse drug reactions, non-reconciled medicines on discharge prescription (resulting in medications being unintentionally continued, changed, stopped or restarted), therapeutic failure (including poor-compliance, poor patient knowledge on medications and reduced dose at discharge) or dispensing errors. We excluded readmissions related to medicines commenced after the patients’ discharge from the study.

We analysed the direct costs of medication related hospital readmissions. We used a tertiary-care hospital cost of 24.60 United States dollars (US$, conversion rate US$ 1 to 130 Sri Lankan rupee in 2014) per bed and day^18^^,^^19^ and multiplied that cost by the estimated number of prevented readmissions in the intervention group and by the observed average duration of a hospital readmission stay. Finally, we extrapolated the estimated cost to a full year.

### Data Analysis

We entered the data into SPSS, Version 21 (IBM, Chicago, USA). An independent second investigator cleaned and audited the entered data. To identify differences between the groups, we used *χ2* test for categorical data and independent sample *t*-test for parametric continuous data. We considered *P*-values less than 0.05 to be statistically significant.

### Ethics

We received ethical approval from the Ethics Review Committee of the Faculty of Medicine, University of Kelaniya, Sri Lanka (Ref. No. P 12/01/2012). The trial was registered under Sri Lanka Clinical Trials Registry (Reg No: SLCTR/2013/029).

We obtained written informed consent, in their own language, from each patient or their relative if the patients were not competent to give consent. The purpose of the trial, the voluntary nature of the consent and the ability of participants to withhold the consent without any effect on their medical care were clearly explained before obtaining consent.

## Results

**Fig. 1:**
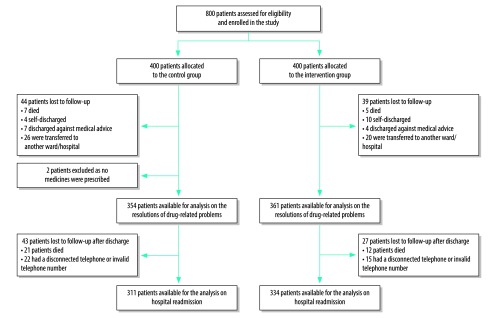
Flowchart of participants included in the non-randomized controlled clinical trial on ward-based pharmacist and hospital readmission, Sri Lanka, 2013–2014

We recruited 800 patients, of them data from 361 patients in the intervention group and 354 patients in the control group were available for analysis of resolution of drug-related problems and appropriateness of medication at discharge. For the assessment of drug-related hospital readmissions, the assessment pharmacist reached and interviewed 334 patients in the intervention group and 311 patients in the control group (Fig. 1).

There were no significant differences between the two groups in age, gender, highest level of education, underlying noncommunicable disease diagnosis and number of medications (Table 1).

**Table 1:** Description of patients participating in non-randomized controlled clinical trial on ward-based pharmacist and hospital readmission, Sri Lanka, 2013–2014

Variables	Control (*n* = 354)	Intervention (*n* = 361)	*P*
**Age in years, mean (SD)**	58.3 (14.8)	56.9 (15.2)	0.270
**Gender, no. (%)**			0.478
Male	171 (48.3)	184 (51.0)	
Female	183 (51.7)	177 (49.0)	
**Education, no. (%)**			0.128
No schooling	6 (1.7)	7 (1.9)	
Grade 1–5	46 (13.0)	39 (10.8)	
Grade 6–11	119(33.6)	114 (31.6)	
Completed ordinary level examination	107 (30.2)	104 (28.8)	
Completed advanced level examination	61 (17.2)	78 (21.6)	
University degree or higher	14 (4.0)	19 (5.3)	
Other	1 (0.3)	0 (0.0)	
**Diagnosis, no (%)**			0.156
Cardiovascular diseases	169 (47.8)	163 (45.2)	
Endocrine diseases	78(22.1)	81(22.3)	
Chronic respiratory diseases	33 (9.4)	27 (7.6)	
Gastrointestinal diseases	20 (5.6)	30 (8.4)	
**No. of medications per patient, mean (SD)**	6.1 (3.0)	6.2 (3.0)	0.578

SD: standard deviation.

### Drug-related problems

The two pharmacists identified in total 1027 drug-related problems in the intervention group, which was similar to the 1217 problems identified in the control group. Resolution of drug-related problem was significantly higher in the intervention group than the control group; 57.6% (592/1027) versus 13.2% (161/1217; *P* < 0.001; Table 2).

**Table 2:** Drug-related problems, medication appropriateness and hospital readmissions for patients with noncommunicable diseases, a non-randomized controlled trial on ward-based pharmacist, Sri Lanka, 2013–2014

Outcome	Control group (354 patients)	Intervention group (361 patients)	*P*
**No. of drug-related problems**	1217	1027	0.25
**No. of resolved drug-related problems (%)**	161 (13.2)	592 (57.6)	< 0.001
**MAI at discharge^a^**			
Mean score per patient (SD)	4.3 (6.5)	1.3 (2.9)	< 0.001
Mean score per medication	0.7 (2.7)	0.2 (1.2)	< 0.001
No. of patients with appropriate medicines at discharge,^b^ (%)	105 (29.7)	202 (56.0)	< 0.001
**Drug-related hospital readmissions**			
No. of patients reached and interviewed^c^	311	334	
No. of drug-related hospital readmissions (%)	93 (29.9)	44 (13.2)	< 0.001
No. of readmissions due to non-compliance to medicines (%)	49 (52.7)	15 (34.1)	0.03
No. of readmissions due to non-reconciliation of medications (%)	17 (18.3)	1 (2.3)	< 0.001

CI: confidence interval; MAI: medication appropriateness index; SD: standard deviation.

^a^ MAI scores range from 0–18 with lowest scores most appropriate.^19^

^b^ We categorized those patients with a score of zero for all their medications as receiving appropriate medications.

^c^ After hospital discharge, the assessment pharmacist interviewed all subjects monthly, for a period of six months, to identify any drug-related hospital readmissions.

The intervention pharmacist prospectively identified 723 drug-related problems in the intervention group. Of these, depending on the nature of the problem, the pharmacist discussed 274 (37.9%) drug-related problems with the health-care team and 449 (62.1%) problems with the patients. The health-care team accepted the pharmacist’s recommendations for 227 (82.8%) drug-related problems, however only 202 recommendations were implemented (Table 3). Table 4 summarizes the number and type of drug-related problems per therapeutic class and Box 1 presents some example of advices the pharmacist gave to patients.

**Table 3:** Outcomes of drug-related problems Sri Lanka, 2013–2014

Group	No. (%)			
	Self-resolved	Resolved^a^	Not resolved	Loss to follow-up^b^
**Control group (1217 drug-related problems)**	161 (13.2)	N/A	1041 (85.5)	15 (1.2)
**Intervention group (1027 drug-related problems)**				
Prospective identification^c^				
Health-care team (274 drug-related problems)^d^	N/A	202 (73.7)	72 (26.3)^e^	N/A
Patient (449 drug-related problems)^f^	N/A	360 (80.2)	65 (14.5)	24 (5.3)
Retrospective identification (304 drug-related problems)^g^	30 (9.9)	N/A	274 (90.1)	N/A

N/A: not applicable.

^a^ The intervention that pharmacist suggested was accepted and implemented.

^b^ Due to death or transfer.

^c^ By clinical intervention pharmacist.

^d^ Intervention pharmacist discussed the problems with the team and gave recommendations about solution to the problems.

^e^ Of the 72 problems that were not resolved, the health-care team accepted but did not implement the pharmacist’s recommendations for 25 problems and 47 recommendations were not accepted.

^e^ Intervention pharmacist discussed the problems with the patients and gave recommendations.

^f^ By assessment pharmacist.

**Table 4:** Types of drug-related problems, per therapeutic class, identified during the non-randomized controlled clinical trial on ward-based pharmacist and hospital readmission, Sri Lanka, 2013–2014

Therapeutic class of medicine	Most commonly prescribed medicines	Type of drug-related problem, no.					
		Adverse drug reaction	Dosing error	Wrong medicine or no medicine	Drug use problem	Interaction	Other^a^
Medicine used for allergy or anaphylaxis	Chlorpheniramine, flunarizine, loratadine, cinnarizine, fexofenadine, cetirizine	0	0	15	0	0	0
Analgesics	Paracetamol, diclofenac, tramadol	5	1	15	1	1	0
Anti-infectives	Co-amoxiclav, amoxicillin, cloxacillin, penicillin, cefuroxime, cefixime, clarithromycin, ciprofloxacin, norfloxacin, metronidazole	4	56	76	9	18	0
Cardiovascular medicines	Atorvastatin, aspirin, losartan, isosorbide mononitrate, furosemide, clopidogrel, glyceryl trinitrate, enalapril, spironolactone, atenolol	163	26	378	88	33	131
Endocrine medicines	Metformin, tolbutamide, insulin, gliclazide, thyroxine, glibenclamide, alendronate sodium	3	13	76	73	34	14
Ear and ophthalmic medicines	Betahistine, prednisolone eye drops, xylometazoline, timolol	0	0	1	1	0	0
Gastrointestinal medicines	Omeprazole, pantoprazole, esomeprazole, famotidine, lactulose, domperidone, ursodiol, *Helicobacter pylori* kit^b^	3	102	125	5	53	0
Genitourinary medicines	Tamsulosin, finasteride, sildenafil	0	2	7	0	0	0
Immunomodulators and antineoplastics	Azathioprine, methotrexate, betamethasone, dexamethasone, prednisolone, sulfasalazine	20	6	8	2	0	0
Neurological medicines	Phenytoin, sodium valproate, carbamazepine, carbidopa/levodopa, benzhexol, gabapentin, pregabalin	0	4	24	3	1	1
Psychotropic medicines	Amitriptyline, fluoxetine, alprazolam, clonazepam, phenobarbital, risperidone, diazepam, olanzapine, zolpidem, aripiprazole	0	1	0	0	6	0
Respiratory medicines	Salbutamol, beclomethasone, fluticasone/salmeterol, theophylline, budesonide/formoterol	0	22	97	13	20	1
Vitamins and mineral supplements	Folic acid, calcium t, vitamin B, iron, multivitamins, vitamin C, 1-alfa-cholecalciferol, vitamin A+D	1	3	70	1	5	1

^a^ Treatment failure, patient dissatisfied with therapy despite taking drug(s) correctly, poor understanding of disease, unclear complaints.

^b^ Patient receives triple therapies for 10–14 days. The treatment regimens are: (i) omeprazole, amoxicillin and clarithromycin for 10 days; (ii) bismuth subsalicylate, metronidazole, and tetracycline for 14 days; and (iii) lansoprazole, amoxicillin, and clarithromycin, which has been approved for either 10 days or 14 days of treatment.

Note: We could not classify 402 drug-related problems into a single therapeutic class.

Box 1 **Example of recommendations the ward-based pharmacist gave to patients with drug-related problems, Sri Lanka, 2013–2014**

**Adverse drug reaction**

Pharmacist provided education on how to recognize or minimize adverse drug reactions. Examples include recognizing hypoglycaemic symptoms with diabetic medications or advice to minimize the risk of bleeding with warfarin.

**Dosing error**

Pharmacist provided education on correct use of dosage measuring device or identifying continued use of previous dose that had been changed at earlier visit.

**Wrong medicine or no medicine**

Pharmacist identified that patient had not received any medicine for the indications or had received prescriptions of the same medication from multiple prescribers, for example diuretics and antibiotics.

**Drug use problem**

Pharmacist educated patient on appropriate use of medication, for example, distinguishing between symptom relief and prophylaxis inhalers in asthma or nitrate-free intervals in ischaemic heart disease. Pharmacist gave information on correct storage of medications, such as insulin and glyceryl trinitrate.

**Drug interaction**

Pharmacist educated patient about food-medicine interactions and when to take medicines in relation to food, such as warfarin or nonsteroidal anti-inflammatory drugs.

**Other**

Pharmacist identified patients with high risk of poor compliance or poor knowledge about prescribed medicines.

### Medication appropriateness index

The mean index score per patient at discharge was significantly lower in the intervention group than the control group (1.3 versus 4.3; *P* < 0.001), resulting in a significantly higher proportion of patients who were discharged with appropriate medication (202/361 in the intervention group versus 105/354 in control group; Table 2).

### Hospital readmissions

Of the 645 patients interviewed, 137 reported drug-related readmissions during the follow-up period. In the control group, 29.9% (93/311) of patients had at least one drug-related hospital readmission, which was significantly higher than for the patients in the intervention group (13.2%; 44/334; *P* < 0.001; Table 2).

#### Estimated savings

Average length of hospital stay for a drug-related readmission was two days. The study pharmacist contributed to the care of eight patients per day and hence they could attend 2500 patients annually. The difference of drug-related hospital readmissions associated with the pharmacist’s intervention was 16.7% (95% confidence interval, CI: 10.5–23.0), resulting in an estimated 108 adverted readmissions during the study period. This reduction would save approximately 835 bed days per year. Estimated direct savings due to reduced readmissions is US$ 20 541 (95% CI: 10 340–24 200), which is greater than an annual pharmacist salary of US$ 2880.^20^

## Discussion

In this study, a ward-based pharmacist intervention was associated with a significant reduction in drug-related hospital readmissions. This reduction was consistent with the observed improvement in resolving drug-related problems during hospital stay and the discharge medication appropriateness index. These improvements are consistent with findings from high-income countries where clinical pharmacy services are well established.^6^^,^^21^^,^^22^ In Sweden, the addition of a clinical pharmacist to the health-care system undertaking similar interventions as in our study reduced hospital readmissions and led to a saving of US$ 1 000 000 per year in health-care costs.^5^ Clinical pharmacists in Australia providing timely communication of the discharge medication plan to patients’ primary-care physicians and community pharmacists led to significantly reduced unplanned hospital readmissions.^6^ A systematic review on economic evaluations of clinical pharmacy services demonstrated cost–effectiveness, however, the review lacked studies from Asia or Africa.^8^ In our study, a simple cost analysis, which did not include estimates of societal costs, supports the introduction of clinical pharmacy service in Sri Lanka.

Lack of provision of patient information on safe medication administration at discharge is a common problem within the Sri Lankan hospital system.^23^ This intervention directly addressed this gap as an intervention pharmacist provided both verbal and written medication information to patients. This communication combined with a higher proportion of appropriate discharge prescriptions were likely to have contributed to reducing drug-related hospital readmissions in the intervention group.

In this study, the proportion of the drug-related problems that were resolved was significantly higher in the intervention group than the control group, which also has been shown in other studies.^5^^,^^23^^,^^24^ While one can assume that resolution of drug-related problems during the inpatient stay may have improved individual patient outcomes, our study was not powered to demonstrate this outcome.

Medical student courses in Sri Lanka teach core elements of quality use of medicines principles, but few institutional resources exist to support quality use of medicines, such as having a clinical pharmacist. This study was not powered to demonstrate improved prescribing behaviour of individual physicians and their ability to anticipate and avoid frequent adverse events. These would be interesting questions for future research.

In Sri Lanka, public hospital pharmacists only dispense medicines to inpatients. This study provides evidence to support the addition of a ward-based clinical pharmacy services for noncommunicable diseases in hospitals. We have previously demonstrated a high acceptability of the pharmacist’s recommendations by the health-care team, especially the physicians.^15^ This supports the feasibility of introducing clinical pharmacists into ward-based health-care teams in Sri Lankan public hospitals. The acceptability is comparable to results from studies conducted in countries where clinical pharmacy is well developed and established.^21^^,^^24^

Given the increasing burden of noncommunicable diseases in low- and middle-income countries,^2^ health systems need to adjust as part of the overall noncommunicable diseases strategy. Furthermore, our findings demonstrate a strategic approach to addressing the World Health Organization’s 3rd Global Patient Safety Challenge of reducing harm from medicines in high risk situations, addressing appropriateness of medicines and reducing risks at transitions of care.^25^

The Sri Lankan government has invested in clinical pharmacy training as a component of a Bachelor of Pharmacy. The government has now the capacity and an opportunity to improve health outcomes by investing in ward-based clinical pharmacy services in public hospitals.^26^ Currently, up to 100 students are graduating from five state universities annually, however, neither health policy nor funded positions has been established for the implementation of clinical pharmacy services in the country. The findings of this study provide evidence to policy-makers to support the establishment of clinical services in Sri Lanka and in other low- and middle-income countries. Our results are widely applicable especially to settings where the clinical pharmacy service is not well established.

The study has some limitations. First, the study population was patients with noncommunicable diseases, thus the results might not be generalizable to other patient groups. While a limited analysis demonstrated cost savings, a more extensive cost analysis in this patient group and other groups should be done to explore the most cost–effective way of using clinical pharmacists in Sri Lanka. Second, the results are likely to be only generalizable to tertiary-care hospitals with strong support from lead medical clinicians. This level of support may not be available in smaller hospitals. Nevertheless, tertiary hospitals can provide training for pharmacists as health professionals in a multidisciplinary care team. These trained clinical pharmacists. could then work in lower level hospitals. Third, doctors being aware of the study may have improved their prescribing behaviour even for the control group, the so-called Hawthorne effect.^27^ However, any improvement in quality use of medicines in the control group, as a result of improved prescribing, would likely reduce the difference between control and intervention outcomes, leading to underestimation of the effect. Fourth, the assessment pharmacist was not blinded to patient group allocation. However, this was not likely to produce significant bias in the objective outcomes of readmission, measurement of medication appropriateness and drug-related problems, which were assessed using established validated tools. Fifth, our intervention pharmacists did receive mentoring via teleconference from Australian clinical pharmacists who were part of the investigation team and the Collaboration of Australian and Sri Lankans for Pharmacy Practice Education and Research. Absence of such support may affect the generalisability of our study. Similar collaborations between pharmacy workforce in low-and middle-income countries and high-income countries would be a strategic component of the initial stages of a scale-up of clinical pharmacy services. Finally, the study used self-reported responses to assess patients’ drug-related hospital readmissions. Self-reporting is a less reliable method than more objective measures. However, the magnitude of the problem would have been the same in both groups.

A collaborative approach to optimizing medicine management with the addition of a ward-based clinical pharmacist is effective in providing overall improvement in quality use of medicines and health outcomes in patients with chronic noncommunicable diseases in a Sri Lankan hospital setting. The results of this trial provide an evidence base for policy-makers in Sri Lanka and other low- and middle-income countries to implement ward-based clinical pharmacy services, using local clinically trained pharmacy graduates.
