# Corrigendum

**DOI:** 10.2471/BLT.18.100518

**Published:** 2018-05-01

In Lelwala Guruge Thushani Shanika, Shaluka Jayamanne, Chandrani Nirmala Wijekoon, Judith Coombes, Dhineli Perera, et al. Ward-based clinical pharmacists and hospital readmission: a non-randomized controlled trial in Sri Lanka. Bull World Health Organ. 2018 March 1; 96 (3):155–64: 

on page 158, third column, the third, fourth and fifth sentences under the “Estimated savings” title should read “The difference of drug-related hospital readmissions associated with the pharmacist’s intervention was 16.7% (95% confidence interval, CI: 10.5–23.0). This reduction would result in an estimated 417 adverted readmissions and would save approximately 835 bed days per year”

